# Co-hydrolysis of hydrothermal and dilute acid pretreated *populus *slurries to support development of a high-throughput pretreatment system

**DOI:** 10.1186/1754-6834-4-19

**Published:** 2011-07-12

**Authors:** Michael H Studer, Simone Brethauer, Jaclyn D DeMartini, Heather L McKenzie, Charles E Wyman

**Affiliations:** 1 Department of Chemical and Environmental Engineering and Center for Environmental Research and Technology, Bourns College of Engineering, University of California Riverside, 1084 Columbia Avenue, Riverside, CA 92507, USA; 2 BESC BioEnergy Science Center, Oak Ridge National Laboratory, Oak Ridge, TN 37831-6422, USA; 3 Current address: Institute of Process Engineering, Swiss Federal Institute of Technology (ETH Zurich), Sonneggstr. 3, CH-8092 Zurich, Switzerland

## Abstract

**Background:**

The BioEnergy Science Center (BESC) developed a high-throughput screening method to rapidly identify low-recalcitrance biomass variants. Because the customary separation and analysis of liquid and solids between pretreatment and enzymatic hydrolysis used in conventional analyses is slow, labor-intensive and very difficult to automate, a streamlined approach we term 'co-hydrolysis' was developed. In this method, the solids and liquid in the pretreated biomass slurry are not separated, but instead hydrolysis is performed by adding enzymes to the whole pretreated slurry. The effects of pretreatment method, severity and solids loading on co-hydrolysis performance were investigated.

**Results:**

For hydrothermal pretreatment at solids concentrations of 0.5 to 2%, high enzyme protein loadings of about 100 mg/g of substrate (glucan plus xylan) in the original poplar wood achieved glucose and xylose yields for co-hydrolysis that were comparable with those for washed solids. In addition, although poplar wood sugar yields from co-hydrolysis at 2% solids concentrations fell short of those from hydrolysis of washed solids after dilute sulfuric acid pretreatment even at high enzyme loadings, pretreatment at 0.5% solids concentrations resulted in similar yields for all but the lowest enzyme loading.

**Conclusions:**

Overall, the influence of severity on susceptibility of pretreated substrates to enzymatic hydrolysis was clearly discernable, showing co-hydrolysis to be a viable approach for identifying plant-pretreatment-enzyme combinations with substantial advantages for sugar production.

## Background

The BioEnergy Science Center (BESC) addresses the challenge of reducing the recalcitrance of biomass, the dominant obstacle to cost-effective production of biofuels, by engineering of plants together with development of advanced biocatalysts to reduce recalcitrance and improve deconstruction [1]. Recent advances in plant genomics have led to large and diverse genome libraries of plant species that can improve our understanding of how individual plant species perform in ethanol-production processes to help guide future development of feedstocks with potentially advantageous characteristics for cellulosic ethanol production. Because reliable methods to characterize recalcitrance of plant cell walls to saccharification do not yet exist, identification of superior biomass species for ethanol production necessitates screening deconstruction of lignocellulosic biomass by pretreatment and subsequent enzymatic hydrolysis. However, final sugar yields depend not only on biomass characteristics but also on their interaction with pretreatment conditions and enzyme formulations. Furthermore, pretreatment is not a single distinct process but varies depending on the chemicals involved (for example, sulfuric acid, ammonia) and the severity used (that is, the combination of pretreatment temperature, reaction time and concentration of chemical). Because different pretreatment methods typically result in different release patterns of compounds that can vary with biomass type, different enzyme formulations and amounts of enzymes must be tested in order to find the lowest cost combinations. To discover the best combinations of biomass types, pretreatment conditions and enzyme formulations, a process is needed that can be used in a high-throughput (HT) device and that is capable of pretreating and enzymatically hydrolyzing large numbers of biomass samples in a semi-automated and cost-effective way.

Conventional laboratory pretreatment, carried out in tubes, mixed reactors or steam guns, with subsequent enzymatic hydrolysis, requires larger amounts of biomass materials than may be available without sacrificing the plants when screening large numbers of biomass candidates. After pretreatment, the solids are separated from the liquid phase and washed, then subjected to enzymatic hydrolysis [2]. The composition of the solids (dry matter and glucan, xylan, mannan, arabinan and galactan) is then determined, and hydrolytic enzymes are added, based on the carbohydrate analysis of the pretreated solids. The wet solids are weighed and transferred to small Erlenmeyer flasks (125 ml), in which the enzymatic hydrolysis is typically performed at a 1% w/w glucan loading. This manual process, with its many complex and time-consuming steps, is very difficult to translate into an automated HT process that lends itself to screening multiple combinations of biomass materials and enzymes using small quantities of these ingredients. It also does not simulate the most attractive commercial operations, for which it is preferable to avoid separation of solids from liquids, in order to reduce capital costs and opportunities for contamination. Therefore, development of screening tools for HT pretreatment and enzymatic hydrolysis to identify biomass variants with reduced recalcitrance has recently attracted interest [3-5].

**Figure 1:**
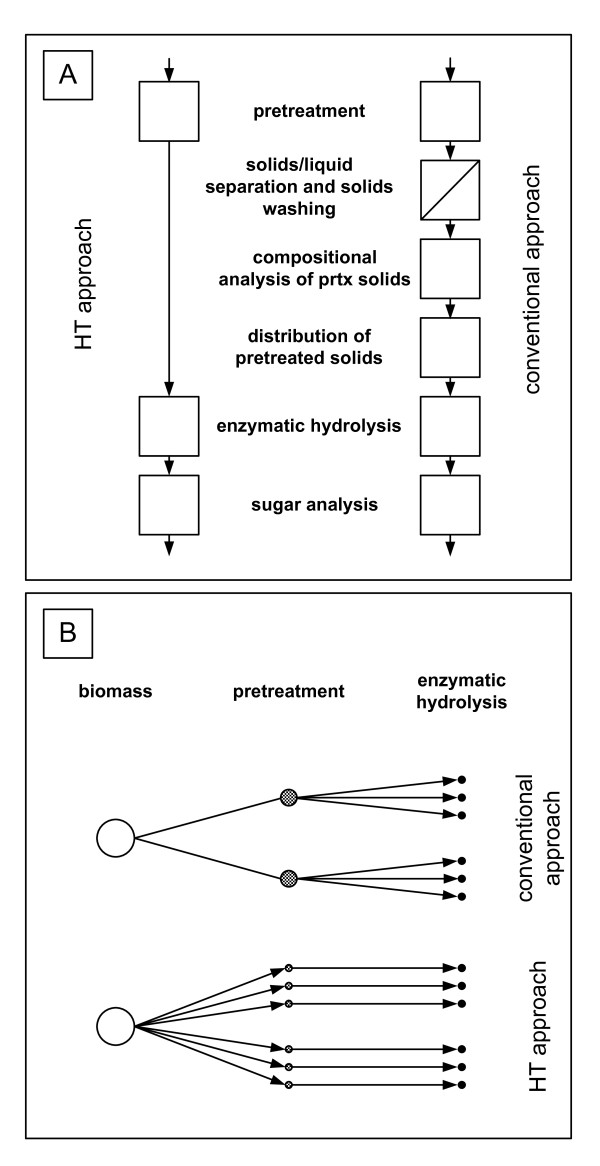
**Schematic illustration of the conventional and the high-throughput (HT) approaches for the analysis of sugar release through pretreatment and enzymatic hydrolysis**. **(A) **Flow diagrams of the conventional and HT approaches for pretreatment, enzymatic hydrolysis and sugar analysis. **(B) **In the HT approach, the same vessel is used for both pretreatment and enzymatic hydrolysis, thereby avoiding processing of biomass between these two operations.

Against this background, we streamlined the pretreatment and enzymatic hydrolysis operations from the large number of conventional steps to a simplified HT process (Figure 1A). In the first step, dry and milled biomass was weighed into each well of custom-made 96-well plates, followed by adding known amounts of water and/or chemicals (for example, sulfuric acid), to each well. The plates were then sealed and heated with condensing steam to the desired pretreatment temperature. We omitted the solid/liquid separation and solids washing steps; instead, the slurry was neutralized using a solution of NaOH, and enzymes were added to the entire pretreated slurry for hydrolysis (Figure 1B), in an approach we term 'co-hydrolysis'. Enzyme loadings were based on the composition (that is, carbohydrate content) of the raw biomass, thus circumventing the necessity for analyzing the pretreated biomass. The custom-made well plate, its mode of operation and its operational reliability have been described previously [5].

In this paper, we discuss the results of the co-hydrolysis process performed with standard laboratory-scale equipment to test whether this unit operation, which underlies all currently discussed HT pretreatment and enzymatic hydrolysis approaches, provides a reasonably accurate measure of advantageous combinations of biomass materials and enzyme formulations. To establish how the performance of co-hydrolysis compares to that for conventional pretreatment, solid-liquid separation, washing of the solids and enzymatic hydrolysis of the washed solids, which we will refer to as 'separate pretreatment and enzymatic hydrolysis' (SPEH), we evaluated sugar release from pretreated biomass for both co-hydrolysis and conventional SPEH approaches using larger standard reactors for each.

To date, systematic investigations of co-hydrolysis, studying the effects of different pretreatment methods and severities, solids concentrations, enzyme dosages, and washing of the pretreated solids on the performance of enzymatic hydrolysis, have been scarce. Researchers have individually investigated the effect of higher solids loadings for non-detoxified pretreated wheat straw at a single enzyme loading [6,7], the effect of washing pretreated solids [8], and the effect of increasing enzyme dosage in comparing washed-solids versus whole-slurry hydrolysis for only one type of solids and enzyme loading [9]. In this paper, we report the combined effects of pretreatment method, severity, enzyme loadings and solids loading on co-hydrolysis performance and its comparison to conventional approaches.

## Results

To test the feasibility of the co-hydrolysis concept as a key feature for HT applications, we compared total sugar yields from separate pretreatment and enzymatic hydrolysis (SPEH) of washed solids with those from co-hydrolysis for several pretreatment and enzymatic hydrolysis conditions. Dilute acid and hydrothermal pretreatments were each performed at two severities with solid substrate loadings as indicated in Table 1. The pretreated material was enzymatically hydrolyzed with cellulase loadings of 15 to 105 mg enzyme protein per gram of substrate (glucan plus xylan) in the raw material, and supplemented with xylanase protein loadings ranging from 5 to 35 mg/g.

**Table 1:** Tested pretreatment conditions^a^

Pretreatment			Solids load, % w/w	Severity
		
Method	Temp, °C	Time, min		
Water only	180	17.6	0.5	Log *R_0_*= 3.6^b^
Water only	180	44.1	0.5	Log *R_0_*= 4.0
Water only	180	17.6	1.0	Log *R_0_*= 3.6
Water only	180	44.1	1.0	Log *R_0_*= 4.0
Water only	180	17.6	2.0	Log *R_0_*= 3.6
Water only	180	44.1	2.0	Log *R_0_*= 4.0
Sulfuric acid 1% w/w	140	10.3	0.5	Log *CS *= 1.5^c^
Sulfuric acid 1% w/w	140	20.5	0.5	Log *CS *= 1.8
Sulfuric acid 1% w/w	140	10.3	2.0	Log *CS *= 1.5
Sulfuric acid 1% w/w	140	20.5	2.0	Log *CS *= 1.8
Sulfuric acid 2% w/w	140	5.1	2.0	Log *CS *= 1.5
Sulfuric acid 2% w/w	140	10.3	2.0	Log *CS *= 1.8

^a^Pretreatment conditions used to compare co-hydrolysis to the conventional characterization method.

^b^, where t denotes time in minutes and T temperature in °C.

^c^*CS *= 10^pH ^× *R_0._*

### Hydrothermal pretreatment

**Figure 2:**
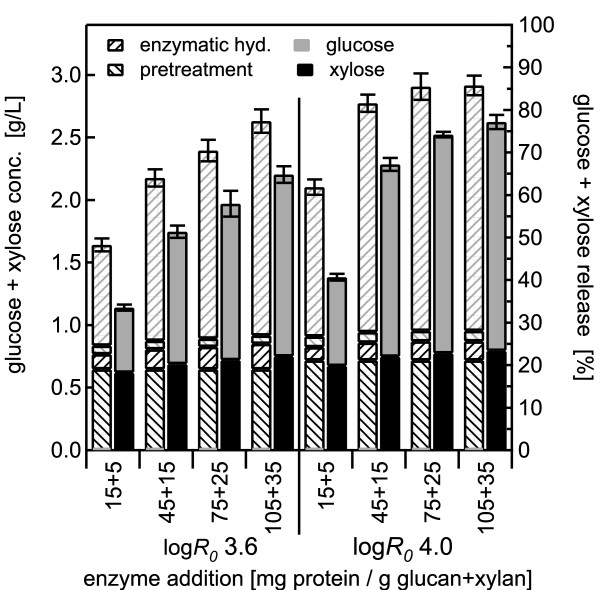
**Hydrothermal pretreatment at 180°C using 0.5% w/w solids concentration**. Glucose and xylose yields from co-hydrolysis compared with those from separate pretreatment and enzymatic hydrolysis (SPEH) for pretreatment of 0.5% w/w *Populus *slurries in water alone at 180°C followed by enzymatic hydrolysis over a range of enzyme loadings from 15 + 5 to 105 + 35 mg of cellulase plus xylanase, respectively, per gram of glucan and xylan in the raw biomass. The eight stacked bars on the left show pretreatment at log *R_0 _*of 3.6 and those on the right at log *R_0 _*of 4.0. The hatched left bar of each immediately adjacent pair shows the distribution of glucose and xylose recovery from pretreatment and enzymatic hydrolysis for the conventional SPEH approach, and the solid bar on the right of each pair presents the amounts of glucose and xylose released from the overall co-hydrolysis method. The error bars represent the standard errors, based on three replicates.

**Figure 3:**
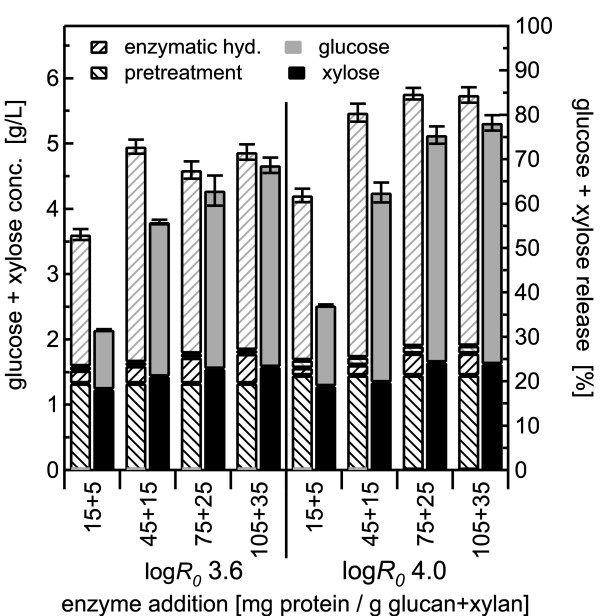
**Hydrothermal pretreatment at 180°C using 1.0% w/w solids concentration**. Glucose and xylose yields from co-hydrolysis compared with those from separate pretreatment and enzymatic hydrolysis (SPEH) for pretreatment of 1% w/w *Populus *slurries in water alone at 180°C followed by enzymatic hydrolysis over a range of enzyme loadings according to the same format as in Figure 2. The error bars represent the standard errors, based on three replicates.

**Figure 4:**
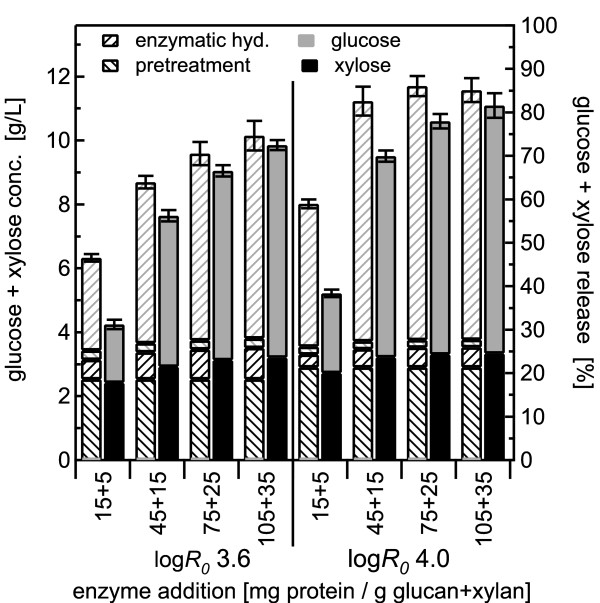
**Hydrothermal pretreatment at 180°C using 2.0% w/w solids concentration**. Glucose and xylose yields from co-hydrolysis compared with those from separate pretreatment and enzymatic hydrolysis (SPEH) for pretreatment of 2% w/w *Populus *slurries in water alone at 180°C followed by enzymatic hydrolysis over a range of enzyme loadings according to the same format as in Figure 2. The error bars represent the standard errors, based on three replicates.

BESC standard poplar was pretreated with water alone at 180°C for 17.6 min and 44.1 min, corresponding to log *R_0 _*severities of 3.6 and 4.0, respectively, with solid loadings of 0.5, 1.0 and 2.0%. Sugar yields from co-hydrolysis were then compared with those from SPEH. Results for the less severe pretreatment conditions are depicted in the left half of the figures (Figure 2, Figure 3, Figure 4), and those from the more optimal pretreatment conditions at higher severity are shown on the right. For each enzyme loading tested, two stacked bars are shown, with the hatched bars on the left representing glucose and xylose yields from the pretreatment and enzymatic hydrolysis steps for SPEH, and the bars on the right representing the combined total sugar yield from co-hydrolysis.

For 0.5% solids (Figure 2), 77% of the xylose was released by pretreatment at log *R_0 _*3.6 and 85% for log *R_0 _*4.0, whereas only about 3% of the total glucose was released at either pretreatment condition, leaving most of the glucose to be digested by the enzymatic hydrolysis. Xylose yields from the enzyme action in the co-hydrolysis method increased with increasing enzyme loading: 12% and 9% of the xylose was released at the highest enzyme loading of 105 + 35 mg/g for both pretreatment severities of log *R_0 _*3.6 and 4.0, respectively, showing that the enzymes released xylose during co-hydrolysis. However, xylose yields from enzymatic hydrolysis in the SPEH method increased with increasing enzyme loadings, from 15% and 13% at the lowest enzyme loading for log *R_0 _*3.6 and log *R_0 _*4.0, respectively, to 24% and 18%, respectively, at the highest enzyme loading. Furthermore, for the lowest enzyme loading (independent of pretreatment severity and solids loading), total xylose yields from co-hydrolysis were lower than those from the pretreatment step alone (before enzymatic hydrolysis) in SPEH (Figure 2, Figure 3, Figure 4). However, with the exception of the 45 + 15 mg/g enzyme loading for the 1% solids pretreatment at log *R_0 _*4.0 (Figure 3), total xylose yields from co-hydrolysis were larger for all other conditions than those from pretreatment alone in SPEH, reaching an increase of 8% and 11% at the highest enzyme loading for the severities of 3.6 and 4.0, respectively, for all solid concentrations.

Glucose yields for co-hydrolysis at 0.5% solids (Figure 2) increased threefold, from 20% at the lowest enzyme loading to 57% at the highest enzyme loading for the lower severity pretreatment, and for the higher severity pretreament, they increased from 28% to 72% for the low and high enzyme loadings, respectively. For SPEH, glucose yields also increased, from 31% to 67%, and from 47% to 77% for the lower and higher severity conditions, respectively.

For both pretreatment severities, total sugar yields from co-hydrolysis and SPEH increased with increasing enzyme loadings until they levelled off at about 70% and 80% for co-hydrolysis and SPEH, respectively, at enzyme loadings of > 75 + 15 mg/g. In spite of the rather high enzyme loadings, corresponding to about 70 FPU/g original glucan in the unpretreated material, the effect of different pretreatment severities was clearly distinguishable, as sugar yields from co-hydrolysis were consistently higher at higher severity and more favourable pretreatment conditions.

**Figure 5:**
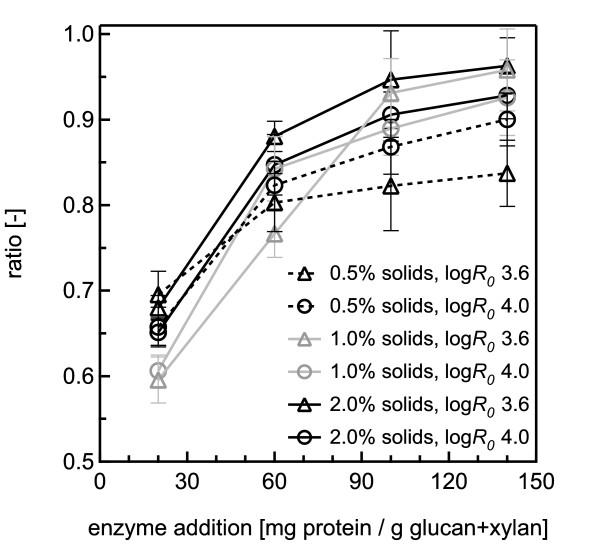
**Ratios of sugar yields from co-hydroysis and SPEH for hydrothermal pretreatments**. Ratios of glucose plus xylose yields from co-hydrolysis to the total yield of these two sugars from separate pretreatment and enzymatic hydrolysis (SPEH) for water-only pretreatment at 180°C according to the severities and solids concentrations noted for each dataset. The error bars represent the standard errors, based on three replicates.

The effect of higher solids concentrations of up to 2% was tested for water pretreatment followed by co-hydrolysis (Figure 3, Figure 4). The results for 0.5%, 1% and 2% solids loading were comparable, showing the same sugar yield patterns from pretreatment and enzymatic hydrolysis for both pretreatment severities. Furthermore, with increasing solids loading, the ratio of xylose release by enzyme action from SPEH to that from co-hydrolysis decreased for the highest enzyme dosage from a factor of 2 for 0.5% solids to 1.5 for 2% solids for both pretreatment severities, whereas the same ratio for glucose remained constant at 1.05 for all severities and solids concentrations. The ratio of glucose plus xylose yields from co-hydrolysis to those from SPEH increased to almost 1 at an enzyme loading of 105 + 35 mg/g, showing that higher enzyme doses could largely overcome whatever inhibitors were reducing enzyme action at lower doses (Figure 5). Furthermore, the ratios decreased with decreasing solids concentrations. For 0.5% solids, the ratios were lower at the lower pretreatment severity, whereas for 1% and 2% solids, the ratios at the lower pretreatment severity were always higher.

### Dilute acid pretreatment

**Figure 6:**
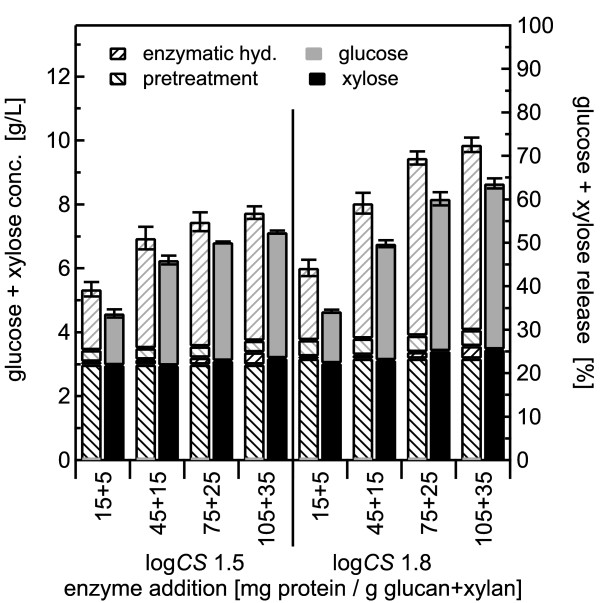
**Dilute acid pretreatment using 1% w/w sulfuric acid with 2.0% w/w solids concentration**. Glucose and xylose yields from co-hydrolysis compared with those from separate pretreatment and enzymatic hydrolysis (SPEH) for pretreatment of 2% w/w *Populus *slurries in 1% w/w sulfuric acid at 140°C followed by enzymatic hydrolysis over a range of enzyme loadings from 15 + 5 to 105 + 35 mg of cellulase plus xylanase, respectively, per gram of glucan and xylan in the raw biomass. The eight stacked bars on the left show pretreatment for log *CS *of 1.5 and those on the right for log *CS *of 1.8. The representation format is the same as that described in Figure 2. The error bars represent the standard errors, based three replicates.

**Figure 7:**
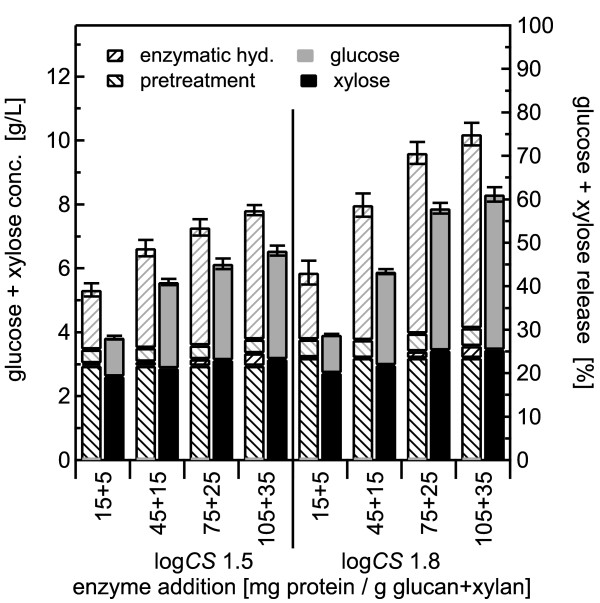
**Dilute acid pretreatment using 2% w/w sulfuric acid with 2.0% w/w solids concentration**. Glucose and xylose yields for co-hydrolysis compared with separate pretreatment and enzymatic hydrolysis (SPEH) for pretreatment of 2% w/w *Populus *slurries in 2% w/w sulfuric acid at 140°C followed by enzymatic hydrolysis over a range of enzyme loadings according to the same format as in Figure 6. The error bars represent the standard errors, based on three replicates.

Initially, 2% slurries of BESC standard poplar were pretreated at 140°C for combined severities of log *CS *1.5 and 1.8 followed by enzymatic hydrolysis. Sulfuric acid concentrations of 1% and 2% were applied to test whether enzyme performance in co-hydrolysis dropped with increases in loadings of acid and of neutralization salts. For 1% sulfuric acid, pretreatment released 89% and 95% of the xylose for the lower and higher severity conditions, respectively (Figure 6). Applying 2% sulfuric acid increased xylose yields from 90% to nearly 100% during pretreatment for the lower and higher severity pretreatment conditions, respectively (Figure 7). Glucose yields from pretreatment alone were low, but increased slightly with severity, from 3% to 5% with 1% sulfuric acid and from 4% to 6% with 2% acid.

For co-hydrolysis following pretreatment with 1% sulfuric acid, adding more enzyme increased xylose yields from essentially 0% to 6% for materials pretreated at log *CS *1.5, and from 0% to 8% for materials pretreated at log *CS *1.8. For 2% acid, increasing amounts of enzyme for co-hydrolysis increased xylose yields from near 0% to 3% for materials pretreated at log *CS *1.5, but had little effect on materials pretreated at higher severity. Adding more enzyme also enhanced xylose release from SPEH, reaching a total xylose yield of essentially 100% for both pretreatment severities and acid concentrations at the higher doses.

For the lower severity pretreatment with 1% acid, increasing overall enzyme loading increased glucose yield after co-hydrolysis from 12% to 35%, versus a corresponding change in glucose yield from 15% to 36% for SPEH. For higher severity pretreatment with 1% acid, increasing enzyme loading increased glucose yield from 11% to 46% for co-hydrolysis, and from 17 to 52% for SPEH. For the 2% sulfuric acid pretreatment, adding more enzyme increased glucose yields for co-hydrolysis from 8% to 29% for the material pretreated at lower severity, and from 6% to 42% for material pretreated at higher severity, versus corresponding increases from 14% to 35% and 15% to 54% for SPEH.

For both pretreatment severities and both acid concentrations, the yields of glucose plus xylose from SPEH increased rapidly with increasing enzyme loadings and changed little between the two highest enzyme loadings (Figure 6, Figure 7), whereas for co-hydrolysis, the yields continued to increase slightly even at high enzyme doses, and in addition, they were somewhat lower than the corresponding yields from SPEH even at very high enzyme loadings. Nonetheless, the trend for increasing yields with increasing severity and enzyme loadings was evident for both co-hydrolysis and the classic SPEH method.

**Figure 8:**
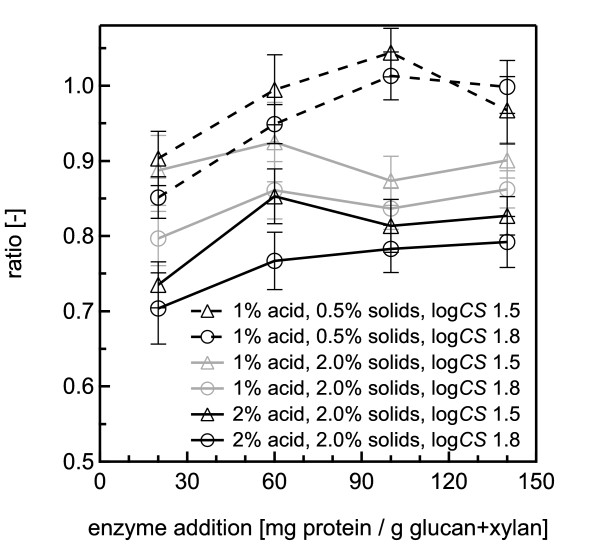
**Ratios of sugar yields from co-hydroysis and SPEH for dilute acid pretreatments**. Ratios of glucose plus xylose yields from co-hydrolysis to the total yield of these two sugars from SPEH for water-only pretreatment at 140°C of 2% w/w *Populus *slurries according to the severities and acid concentrations noted for each dataset. The error bars represent the standard errors, based on three replicates.

For low-severity pretreatment with 1% sulfuric acid, there was no clear trend in the relationship of the ratios of the total sugar yields from co-hydrolysis to those from SPEH with increasing enzyme loading, with the ratio being held fairly constant at around 0.9 (Figure 8). However, for the high-severity condition with 1% acid and for both pretreatment severities with 2% acid, the ratio of the yields increased more noticeably with increasing enzyme loading. In addition, the ratios decreased with increasing pretreatment severity and acid concentration. Overall, differences between the total sugar yields from co-hydrolysis and those from SPEH were more pronounced at the higher severity pretreatments and higher acid concentrations.

**Figure 9:**
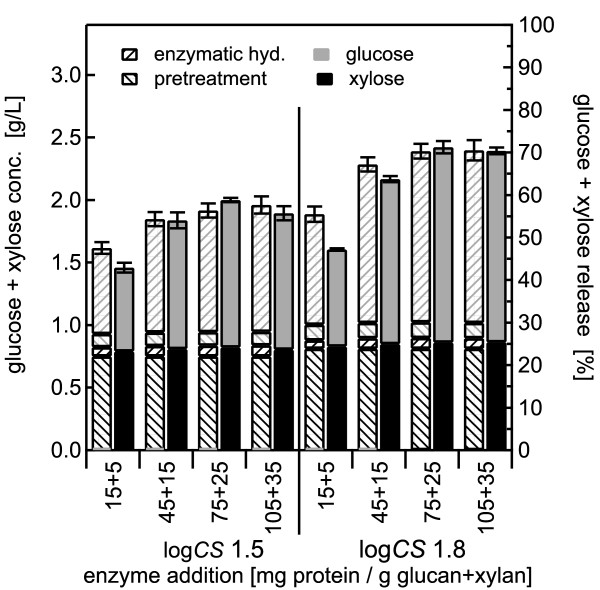
**Dilute acid pretreatment using 1% w/w sulfuric acid with 0.5% w/w solids concentration**. Glucose and xylose sugar yields from co-hydrolysis compared with those from separate pretreatment and enzymatic hydrolysis (SPEH) for pretreatment of 0.5% w/w *Populus *slurries in 1% w/w sulfuric acid at 140°C followed by enzymatic hydrolysis over a range of enzyme loadings according to the same format as in Figure 6. The error bars represent the standard errors, based on three replicates.

Because of the high inhibition seen with dilute acid co-hydrolysis compared with SPEH, we also conducted experiments with 0.5% solids concentrations and 1% sulfuric acid. The differences between co-hydrolysis and SPEH were reduced considerably, and only became appreciable at the lowest enzyme loading (Figure 8, Figure 9).

## Discussion

The prerequisite for successful screening of biomass types for reduced recalcitrance is the transferability of results obtained by co-hydrolysis to results from SPEH. We therefore compared the sugar release from poplar wood between co-hydrolysis and SPEH as a function of solids concentration and enzyme loading for two different pretreatments at each of two severities.

### Hydrothermal pretreatment

At a single pretreatment condition, any differences in total sugar yield between co-hydrolysis and pretreatment coupled with washed solids hydrolysis (SPEH) could only result from differences in yields from enzymatic hydrolysis, and consequently, total yields from co-hydrolysis would be expected to be no lower than those from pretreatment alone. However, at the lowest enzyme loading, the total xylose yields from co-hydrolysis were lower than those from pretreatment alone before the hydrolysis of the washed-solids in SPEH, whereas at the highest enzyme loading, the total xylose yields from co-hydrolysis were lower than those from pretreatment alone before SPEH. Because a large fraction of the xylose in solution after pretreatment is oligomeric after water-only pretreatment, post-hydrolysis was used to measure the solubilized fraction [10]; however, this method is subject to some errors that might overcompensate for degradation during post-hydrolysis [11]. In addition, low amounts of xylanase seemed to be unable to hydrolyze all oligomers to monomers during co-hydrolysis, reducing the amount of xylose detected by HPLC. Further work is needed to understand the cause of these differences in xylose yields, as they could account for some portion of the higher xylose yields from the SPEH procedure.

Less than 4% of the glucan was solubilized during pretreatment for the hydrothermal conditions tested, leaving most of it requiring release during enzymatic hydrolysis. For co-hydrolysis, yields at the highest enzyme loading were 3 times higher than those at the lowest, whereas for SPEH, they were only 1.5 times higher. Furthermore, glucose release was 50% lower from co-hydrolysis at low enzyme loadings than from SPEH. However, higher enzyme loadings almost completely overcame the difference in glucan yields between co-hydrolysis and SPEH, indicating strong enzyme inhibition. Ratios of glucose plus xylose yields for co-hydrolysis to those from SPEH showed lower inhibition for lower-severity pretreatment. Interestingly, the ratios were always higher for higher solids concentrations, possibly because release of inhibitors did not increase with solids loadings over the range studied, whereas the total mass of added enzyme increased with increasing solids concentrations. Studies of inhibition of enzymatic hydrolysis are scarce, but possible inhibitors produced from water-only pretreatment include sugar-degradation products such as 5-hydroxymethylfurfural and furfural, or soluble products such as acetic acid released from biomass [12], lignin-degradation products [13,14], and glucose and xylose oligomers [15]. Thus, although the causes for the observed differences in glucose yield between co-hydrolysis and SPEH merit further investigation, the yield trends for both approaches clearly showed the effect of pretreatment severity and enzyme loadings on sugar release.

### Dilute acid pretreatment

Dilute sulfuric acid pretreatment solubilized almost all of the xylan, and 2% acid further increased xylose yields, even though the severity remained constant. Glucose release increased slightly with higher-severity pretreatment, and 2% sulfuric acid released slightly more glucose than did 1% acid. Dilute acid gave a maximum glucose yield from pretreatment of 6% for the time span covered, twice the value of that from hydrothermal pretreatment.

For the conditions tested here, glucose yields from enzymatic hydrolysis of washed poplar solids after dilute acid pretreatment were generally lower than for washed solids after hydrothermal pretreatment. However, as with hydrothermal pretreatment, higher enzyme loadings and higher pretreatment severity increased sugar yields for dilute acid pretreatments under both hydrolysis regimens. Because lower enzyme loadings did not show this trend for co-hydrolysis, enzyme loadings of > 75 + 25 mg/g were needed to obtain identical glucose yield trends from both methods at 2% solids concentrations. However, reducing the solids concentrations to 0.5% resulted in similar yields for co-hydrolysis and SPEH for all but the lowest enzyme loading of 15 + 5 mg protein per gram glucan plus xylan. Furthermore, yield differences resulting from pretreatment severity were still discernable at this lower loading.

Yields were highest for 2% sulfuric acid-pretreated materials for the enzymatic hydrolysis of washed solids but were highest for 1% acid-pretreated materials for the co-hydrolysis runs. This finding suggests that salt loadings from acid neutralization, compounds released from biomass by pretreatment, or compounds formed during pretreatment increased with acid concentration. It is notable that glucose yields at low enzyme loadings were as low as 6% for co-hydrolysis at 2% solids concentration after pretreatment at high severity with 2% acid, whereas glucose yields reached 26% with lower solids loading and acid loading of 0.5% and 1%, respectively.

Comparing total sugar yields from co-hydrolysis with those from SPEH found that yield differences became more pronounced at higher severities, solids loadings and acid concentrations. Higher enzyme loadings could partly overcome enzyme inhibition, but the yield differences between co-hydrolysis and washed solids hydrolysis remained. Fortunately, operation with a lower solids loading of 0.5% largely overcame these differences even at low enzyme loadings.

## Conclusions

Co-hydrolysis achieved good yields of glucose and xylose for poplar slurries at solid concentrations up to 2% for pretreatment with water alone, and at sulfuric acid concentrations of 1% and 2%. However, protein loadings in the range of 100 mg of xylanase plus cellulase per gram of glucan plus xylan in the original biomass were needed to achieve yields from co-hydrolysis similar to those from SPEH for hydrothermal pretreatment of poplar. Furthermore, high enzyme loadings could not fully compensate for dilute sulfuric acid pretreatment at 2% solids concentration, apparently due to greater release and/or generation of inhibitors, but operation with 0.5% solids resulted in identical performance between co-hydrolysis and SPEH. In addition, even when yields were somewhat lower for co-hydrolysis than for SPEH, the influence of pretreatment severity on enzymatic hydrolysis of the pretreated substrate was still clearly discernable for pretreatment with water alone and with dilute sulfuric acid, provided enough enzyme was used. Thus, co-hydrolysis is viable for initial screening of plants to identify those that are less recalcitrant to sugar release through pretreatment and enzymatic hydrolysis. It can also help determine whether lower severity pretreatments could be used to achieve similarly high sugar yields, determine enzyme formulations that promote sugar release, and facilitate the identification of enzymes that can withstand inhibitors produced in biomass pretreatment. It is important to note that this screening tool can identify substrate-pretreatment-enzyme combinations that could simplify commercial operations by avoiding the need for hydrolyzate removal before enzymatic hydrolysis.

## Methods

### Biomass

A genotype of *Populus trichocarpa *grown at the Oak Ridge National Laboratory (termed BESC standard poplar in this paper) was used for all experiments. The logs were debarked, split with an axe, chipped (Yard Machines 10HP, MTD Products Inc., Cleveland, OH, USA) and knife-milled (Model 4 Wiley Mill, Thomas Scientific, Swedesboro, NJ, USA) through a 1 mm screen. The wood was air-dried in Colorado at the National Renewable Energy Laboratory for approximately 1 month until it reached a moisture content of 6.67 ± 0.08% w/w. All material was then sieved to less than 20 mesh (< 0.85 mm) and greater than 80 mesh (> 0.180 mm) (Ro-Tap RX-29, W.S. Tyler, Mentor, OH, USA). Particles larger than 20 mesh were reground and sieved again, and the resulting 20 to 80 mesh fraction was mixed with the 20 to 80 fraction obtained originally. The BESC standard poplar contained 46.2% w/w glucan and 14.8% w/w xylan.

### Pretreatments

Hydrothermal pretreatments were performed with solid loadings of 0.5%, 1% and 2% w/w, and dilute sulfuric acid pretreatments with 0.5% and 2% w/w solids concentrations, with each concentration based on the mass of raw biomass before pretreatment. Dilute acid pretreatments were carried out at acid concentrations of 1% and 2% w/w based on the total liquid phase (including the water contained in the biomass) and at a temperature of 140°C, whereas hydrothermal pretreatments were carried out at 180°C. Pretreatment severities were calculated as defined by Chornet and Chum for hydrothermal and dilute acid pretreatments, respectively [16,17]. Table 1 summarizes the conditions applied and the calculated severities for the pretreatments reported. Pretreatments were conducted in a 1 litre stirred tank reactor made of Hastelloy (4520 Series; Parr Instruments, Moline, IL, USA) equipped with a double- stacked pitch-blade impeller (∅ = 50 mm). The stirring rate was set to 100 rpm, and the agitator rotated in a direction to push the contents downward. The reactor was heated in a fluidized sand bath, in which the temperature was set to 320 and 400°C, for the dilute acid and hydrothermal pretreatments, respectively. The target temperature was maintained by floating the reactor a small distance above the undulating surface [18]. The timer was started when the reaction temperature was reached (± 1.5°C, the tolerance of the Type K thermocouple used). The heat-up and cool-down times were about 3 and 2 minutes, respectively, for all pretreatments.

### Enzymatic hydrolysis

After pretreatment, 25 ml aliquots were removed from the well-stirred slurry using a 25 ml pipette with the tip cut to produce an opening with an inner diameter of 5 mm. Half of the samples were directly transferred to 125 ml screw cap Erlenmeyer flasks, and the other half to 50 ml centrifuge tubes. The latter fraction was washed three times by centrifugation, decantation and resuspension to 50 ml with deionized water. After washing, the samples were resuspended with deionized water to the original weight, and transferred to 125 ml Erlenmeyer flasks. The supernatant of the pretreated biomass slurries was post-hydrolyzed to determine the total xylose and glucose amounts recovered, using to the standard National Renewable Energy Laboratory method [19]. After pretreatment with dilute sulfuric acid, biomass slurries were titrated to pH 5 with 50% NaOH. To all samples, 1.25 ml of citric acid buffer (pH 4.95) was added to achieve a final concentration in the slurry of 0.05 mol/l, then, 0.25 ml of sodium azide (0.1 g/L) and the appropriate amount of enzyme mixture were added. Cellulase (Spezyme CP, protein content 116.0 mg/ml, lot number 3016295230; Genencore, Palo Alto, CA, USA; Genecnore) and xylanase (Multifect Xylanase, protein content 56.6 mg/ml, lot number 4900667792; Genencore) were mixed at a ratio of 3:1 based on their protein content, and diluted 1:3 with HPLC-grade water. All of the resulting samples were incubated at 50°C in a shaking incubator at 150 with a throw of 25 mm (Multitron 2, Infors-HT, Bottmingen, Switzerland) for 72 hours. All enzymatic hydrolysis experiments were carried out in triplicate.

### Sugar analysis

Glucose and xylose concentrations were analyzed using HPLC. A separation column (Aminex HPX-87H; BioRad, Hercules, CA, USA) with 0.005 mol/l sulfuric acid as the eluent was used in isocratic mode at 65°C on a separation module (Alliance 2695; Waters, Milford, MA, USA) equipped with a refractive index detector (model 2414; Waters) set to 35°C.

## List of abbreviations used

BESC: BioEnergy Science Center; HT: high-throughput; SPEH: separate pretreatment and enzymatic hydrolysis

## Competing interests

CEW is cofounder of Mascoma Corporation and chair of their Scientific Advisory Board. CEW is also member of the Scientific Advisory Board of Mendel Biotechnology, Inc. CEW is also founding Editor in Chief of *Biotechnnology for Biofuels*.

## Authors' contributions

MHS designed and performed the research, analyzed the data and wrote the paper. SB, JDD and HLM participated in performing the experiments and writing the paper. CEW coordinated the research and helped to finalize the manuscript. All authors read and approved the final manuscript.
